# Selective Esophagogastric Devascularization in the Modified Sugiura Procedure for Patients with Cirrhotic Hemorrhagic Portal Hypertension: A Randomized Controlled Trial

**DOI:** 10.1155/2020/8839098

**Published:** 2020-12-05

**Authors:** Yawu Zhang, Lingyi Zhang, Mancai Wang, Xiaoling Luo, Zheyuan Wang, Gennian Wang, Xiaohu Guo, Fengxian Wei, Youcheng Zhang

**Affiliations:** ^1^Department of General Surgery, Lanzhou University Second Hospital, Lanzhou 730030, China; ^2^Hepato-Biliary-Pancreatic Institute, Lanzhou University Second Hospital, Lanzhou 730030, China; ^3^Gansu Provincial-Level Key Laboratory of Digestive System Tumors, Lanzhou 730030, China; ^4^Department of Hepatology, Lanzhou University Second Hospital, Lanzhou 730030, China

## Abstract

**Aim:**

Portal hypertension is a series of syndrome commonly seen with advanced cirrhosis, which seriously affects patient's quality of life and survival. This study was designed to access the efficacy and safety of selective esophagogastric devascularization in the modified Sugiura procedure for patients with cirrhotic hemorrhagic portal hypertension.

**Methods:**

Sixty patients with hepatitis B cirrhotic hemorrhagic portal hypertension and meeting the inclusion criteria were selected and randomly divided by using computer into the selective modified Sugiura group (sMSP group, *n* = 30) and the modified Sugiura group (MSP group, *n* = 30). The primary endpoint measurement is the postoperative rebleeding rate. Secondary endpoint measurements included free portal venous pressure, liver Child–Pugh score, liver volume, portal vein width and blood flow velocity, survival rate, quality of life, and dysphagia as well as other complications one year postoperatively. This trial is registered with ChiCTR, number ChiCTR2000033468.

**Results:**

There was no statistically significant difference in rebleeding rates within one year after surgery between patients in the sMSP and MSP groups (*χ* = 0.11, *p*=0.73). In comparison with the MSP group, the Child–Pugh score of liver function in the sMSP group significantly increased (*χ* = 6.4, *p*=0.04) and the incidence of dysphagia was significantly reduced (*χ* = 6.23, *p*=0.01) one year after surgery. There was a statistically significant difference in the quality of life between the two groups. However, there were no statistically significant differences in free portal venous pressure (MD = −3.44, 95% CI: −7.87 to 0.98, *p*=0.12), postoperative liver volume (3 months: MD = -258.81, 95% CI: −723.21 to 205.57, *p*=0.24; 1 year: MD = −320.12, 95% CI: −438.43 to 102.78, *p*=0.16), postoperative portal vein width (3 months: MD = −0.06, *p*=0.50; 1 year: MD = 0.17, *p*=0.21), portal vein flow velocity (3 months: MD = 1.64, *p*=0.21; 1 year: MD = −1.19, *p*=0.57), 1-year survival rate (*χ* = 1.01, *p*=0.31), and other complications between the two groups.

**Conclusions:**

Selective esophagogastric devascularization in the modified Sugiura procedure may not lower the incidence of rebleeding in the short term based on our findings. However, it may significantly improve quality of life of patients with cirrhotic hemorrhagic portal hypertension, improve liver function, and reduce postoperative dysphagia.

## 1. Introduction

Portal hypertension is a common clinical syndrome characterized by an increase in pressure gradient between the portal vein and the inferior vena cava. Chronic liver diseases including alcoholic or viral cirrhosis are the main causes of portal hypertension [1]. Complications of portal hypertension include esophageal-gastric varices, upper gastrointestinal bleeding, splenomegaly, hypersplenism, ascites, and hypoproteinemia. In particular, upper gastrointestinal bleeding caused by varicose veins is a main course of death in patients with portal hypertension [2, 3]. The risk of gastrointestinal bleeding in patients with severe varices is approximately 30% in 2 years [4], and the risk of rebleeding within 2 years increases to nearly 70% without prophylactic treatment [5]. The mortality rate of patients with esophagogastric variceal bleeding has reduced significantly in recent decades. Nevertheless, the 6-week mortality rate remains as high as 20% [6].

Currently, there are various treatment strategies for portal hypertension and related complications, including medical treatment, endoscopic treatment, interventional therapy, and surgery [6, 7]. In general, patients with portal hypertension and mild or moderate esophageal-gastric varices are preferentially treated medically or with endoscopic ligation in conjunction with sclerotherapy. These treatments have definite curative effects, but approximately 20%–30% of patients still require interventional therapy or surgery [8]. Transjugular intrahepatic portal systemic shunt (TIPS) is currently the most widely used method for the treatment of portal hypertension [9]. In comparison with medical and endoscopic treatment, TIPS can control bleeding more effectively and in a timely fashion, but it cannot reduce mortality rate. Furthermore, TIPS may increase the incidence of hepatic encephalopathy [5, 10]. Liver transplantation is highly recognized as the most effective method for treating portal hypertension in cirrhosis, but is limited by the scarcity of donors and high medical costs [4].

Portosystemic shunting and devascularization are the most commonly used surgical procedures for the treatment of portal hypertension. The former may further increase the risk of liver damage and hepatic encephalopathy due to decreased hepatic blood flow through the portal vein [4]. The Hassab procedure and modified Sugiura procedure are currently the two most important devascularization methods. Studies have shown that the modified Sugiura procedure may be more effective in preventing bleeding [2]. In China, a large number of patients with portal hypertension undergo devascularization surgery. However, complications such as rebleeding due to persistent high pressure of the portal vein have not been effectively solved. To lower postoperative portal pressure and reduce postoperative complications, Yang et al. [11] presented their experience with selective devascularization of the left gastric vein during esophagogastric devascularization, where they achieved satisfactory results. In this study, we conducted selective esophagogastric devascularization in the modified Sugiura procedure to evaluate the efficacy and safety of this procedure.

## 2. Methods

This study was a single-center, randomized controlled trial to evaluate the efficacy and safety of selective esophagogastric devascularization in the modified Sugiura procedure. Selective esophagogastric devascularization is to preserve the esophageal branch of the left gastric vein, or the paraesophageal vein, and to selectively devascularize the lower esophagus perforating vein and gastric branches of the left gastric vein. The study was approved by the Ethics Committee of the Second Hospital of Lanzhou University, and informed consent was obtained from all participating patients. The entire trial followed the guidelines for good clinical practice in clinical trials.

### 2.1. Study Subjects

We recruited patients with cirrhotic hemorrhagic portal hypertension who were scheduled to undergo surgery at the Second Hospital of Lanzhou University between January 2014 and December 2017. Inclusion criteria were as follows: (1) patients with decompensated hepatic cirrhosis after hepatitis B, combined with splenomegaly, hypersplenism, and esophagogastric varices; (2) after the first or second esophageal and gastric variceal bleeding, and the bleeding was stopped by medical treatment for at least one week; (3) no history of endoscopic ligation or sclerotherapy prior to surgery, and no TIPS or other devascularization treatment; (4) no thrombosis in the portal vein and its branches by preoperative abdominal CT or ultrasound; (5) no liver cancer or other malignant tumors were found; (6) it was confirmed during the operation that esophageal veins did not directly enter the esophagus, and perforating veins clearly existed; and (7) adult patients who agreed to undergo the modified Sugiura procedure and sign informed consent.

### 2.2. Study Design

This study used a randomized study design, with the random sequence generated by using a computer. Patients who met inclusion criteria were randomly assigned to the selective modified Sugiura surgery group (sMSP group) or the modified Sugiura procedure group (MSP group) in a 1 : 1 ratio. Random assignment was performed after it was determined that the patient's esophageal vein did not directly enter the esophagus.

### 2.3. Intervention Strategies

#### 2.3.1. Modified Sugiura Procedure [2] (Figure 1)

Under general anesthesia in the supine position, an “L”-shaped incision was made at the ventral midline along the left rib margin. We (1) searched for and ligated the splenic vessels and performed splenectomy; (2) isolated the gastric proximal end close to the greater and lesser curvatures of the stomach and then disconnected and ligated the left gastric vein, posterior gastric vein, short gastric vein, left inferior phrenic vein, and accompanying arteries; (3) dissected the esophagus about 7 cm away from the cardia and disconnected, dissected, and ligated the esophageal vein, high esophageal branch, and esophageal perforating vein; (4) made a 2 cm vertical incision on the anterior gastric wall at the site of 3–5 cm away from the cardia and free of blood vessels; (5) used a #26 size tubular stapler for transesophageal transection anastomosis, approximately 3–4 cm away from the cardia. The vertical incision of the stomach was sutured. After it was determined that there was no active hemorrhage, a drainage tube was inserted and the abdominal cavity was sutured.

#### 2.3.2. Selective Modified Sugiura Procedure (Figure 2)

Based on the modified Sugiura procedure, the left gastric vein and its branches were selectively treated [11]. Namely, the left gastric vein and its esophageal vein branch were saved; only the left gastric vein and the esophageal perforating vein were disconnected and ligated. The remaining procedures were identical to the modified Sugiura procedure.

#### 2.3.3. Others

This mainly refers to patients with paraesophageal veins directly inserting into the esophagus. Those patients were excluded, and the paraesophageal veins inserting into the esophagus were directly disconnected and ligated during the operation. The remaining interventions were the same as described above. All patients were subcutaneously given low molecular weight heparin sodium 1 vial, Qd, on the second day after surgery. When the platelets normalized postoperatively, 100 mg of aspirin was administered orally daily.

### 2.4. Follow-Up

Follow-up was scheduled at 1, 3, 6 months, and 1 year after surgery. Follow-up measurements included routine blood examination, liver function tests, color Doppler ultrasound of the portal vein and hepatic artery, venous color Doppler ultrasound, abdominal CT, quality of life assessment, and complications.

### 2.5. Endpoint Measurements

The primary endpoint measurement is postoperative rebleeding rate. Secondary endpoint measures included free portal venous pressure (FPP), liver Child–Pugh score, liver volume, width of portal vein and blood flow velocity, quality of life, one-year survival rate, and various complications (e.g., hepatic encephalopathy, postoperative abdominal bleeding, portal vein thrombosis, and swallowing difficulty).

For rebleeding, the main postoperative manifestations were hematemesis or hematochezia, diagnosed by gastroscopy. Free portal venous pressure (FPP) was assessed as follows: after laparotomy, FPP was measured three times before splenectomy, after splenectomy, and after transesophageal anastomosis. An indwelling needle with a diameter of 0.7 mm was used to puncture and fix the right gastroepiploic vein, which was about 10–15 cm away from the cardia. The indwelling needle was connected to a detector (Hewlett-Packard, USA) with sensors (Biosensors International, Singapore). Quality of life one year postoperatively was measured by the SF-36 scale. Liver volume was calculated based on abdominal enhanced computational tomography (CT) and measured by the IQQA(R)-Liver Image Analysis System. Portal vein ultrasound was used to measure portal vein width and thrombus.

### 2.6. Statistical Analysis

Statistical analysis was performed using SPSS version 18.0 software, and all analyses were intention-to-treat (ITT) analysis. The *t*-test was used for continuous variables, and the chi-squared test was used for the two categorical variables. The difference between groups was considered statistically significant at a *p* value of less than 0.05.

## 3. Results

### 3.1. Demographic Characteristics

In this study, 75 patients with decompensated cirrhotic hepatitis B were originally recruited. Fifteen patients who did not meet inclusion criteria were excluded, and the remaining 60 patients were randomly assigned to the selective modified Sugiura procedure group (sMSP) (*n* = 30) or modified Sugiura procedure group (MSP) (*n* = 30) (Figure 3). There was no statistically significant difference in baseline characteristics between the two groups (Table 1). The average age of the patients was 45.64 ± 10.49 years, and the male-to-female ratio was 44 : 16. The mean follow-up time was 14.3 ± 5.5 months in the sMSP group and 13.9 ± 4.7 months in the MSP group. One patient in the sMSP group died during the follow-up period due to esophageal venous rebleeding 3 months after surgery; the remaining patients were followed up for at least 1 year.

### 3.2. Primary Endpoint Measurement

#### 3.2.1. Rebleeding Rates

None of the included patients developed esophagogastric hemorrhage during postoperative hospitalization. Six weeks after surgery, two patients in the sMSP group developed hematemesis and melena; gastroscopy diagnosed esophageal and gastric variceal rebleeding; four patients in the MSP group developed rebleeding. In the sMSP group, five patients had esophageal and gastric fundus hemorrhage within 1 year after surgery, of which one patient died of rebleeding 3 months after surgery. The remaining patients were treated with medical or endoscopic therapy with much improvement. The rebleeding rate was also 16.7% (5/30). A total of six patients in the MSP group developed esophagogastric hemorrhage within 1 year after surgery. The rebleeding rate was 20.0% (6/30). No death was reported. There was no statistically significant difference in rebleeding rates between the two groups 1 year after surgery (*χ* = 0.11, *p*=0.73).

### 3.3. Secondary Endpoint Measurements

#### 3.3.1. FPP

In the sMSP group, the FPP value was significantly lower after splenectomy (20.01 ± 4.50 mmHg) and esophageal transection (19.40 ± 4.40 mm Hg) than before splenectomy (27.00 ± 6.41 mmHg). There was a statistically significant difference between the two groups (MD = 6.75, 95% CI: 3.18–10.31, *p* < 0.001; MD = 7.52, 95% CI: 3.93–11.12, *p* < 0.001); The FPP value decreased slightly after transesophageal esophagectomy in comparison to after splenectomy; this difference was not statistically significant (MD = 0.77, 95% CI: −2.13 to 3.691, *p*=0.59).

In the MSP group, the changes in the patient's FPP value were same as the sMSP group (Figure 4). There was no statistically significant difference in FPP values between the sMSP group and the MSP group after esophageal transverse anastomosis (MD = −3.44, 95% CI: −7.87 to 0.98, *p*=0.12).

#### 3.3.2. Child–Pugh Score of Liver Function

There was no statistically significant difference in the Child–Pugh score of liver function between the two groups prior to surgery (Table 1). One year after surgery, the Child–Pugh scores of the patients in the sMSP group were as follows: grade *A*, 7 cases; grade *B*, 19 cases; and grade *C*, 3 cases. The Child–Pugh scores of the patients in the MSP group were as follows: grate *A*, 3 cases; grade *B*, 16 cases; and grade *C*, 11 cases. The differences between the two groups were statistically significant (*χ* = 6.4, *p*=0.04).

#### 3.3.3. Liver Volume

There was no significant difference in liver volume between patients in the two groups before surgery (Table 1). The study results showed that the liver volume of patients in the two groups increased after surgery, but there was no significant difference in liver volume 3 months or 1 year postoperatively between the two groups (MD = −258.81, 95% CI: −723.21 to 205.57, *p*=0.24; MD = −320.12, 95% CI: −438.43 to 102.78, *p*=0.16) (Figure 5).

#### 3.3.4. Width of Portal Vein and Blood Flow Velocity

There was no significant difference in the width of portal vein between patients in the two groups before surgery (Table 1). There was no significant change in the width of the portal vein between patients in the two groups at 3 months and 1 year after surgery (1.29 ± 0.16 vs. 1.36 ± 0.32; 1.40 ± 0.34 vs. 1.22 ± 0.26). There was no statistically significant difference between the two groups (MD = −0.06, *p*=0.50; MD = 0.17, *p*=0.21). No statistically significant difference in portal vein flow velocity was observed between the two groups before surgery (Table 1). There was no statistically significant change in portal vein flow velocity between patients in the two groups at 3 months and 1 year after surgery (12.64 ± 3.98 vs. 10.99 ± 2.35; 12.28 ± 4.37 vs. 13.47 ± 4.80). No statistically significant difference was shown between patients in the two groups (MD = 1.64, *p*=0.21; MD = −1.19, *p*=0.57).

#### 3.3.5. Quality of Life

The SF-36 table was used to evaluate quality of life 1 year after surgery regarding physical functioning, role-physical, bodily pain, general health, vitality, social functioning, role-emotional, and mental health. Patients in the sMSP group had significant improvement in physiologic function (*p* < 0.001), general health (*p* < 0.001), social function (*p* < 0.001), and mental health (*p* < 0.001) in comparison with those in the MSP group. The differences between the two groups were statistically significant (Table 2).

#### 3.3.6. One-Year Survival Rate

One patient in the sMSP group died of esophageal and gastric variceal rebleeding 3 months after surgery. The 1 year survival rate was 96.66%. There were no deaths reported in the MSP group. No statistically significant difference between the two groups was observed (*χ* = 1.01, *p*=0.31). In addition, in those patients who did not meet the inclusion criteria, one patient died of a chest infection following postoperative esophageal anastomotic fistula. The patient was treated with endoscopic sclerotherapy for esophageal and gastric variceal bleeding 1 month before surgery.

#### 3.3.7. Other Complications

In addition to hemorrhage after surgery, postoperative complications also included ascites, portal vein thrombosis, splenic vein thrombosis, dysphagia, abdominal bleeding, abdominal infection, esophageal anastomotic leakage, and hepatic encephalopathy (Table 3). In particular, in comparison with the sMSP group, the proportion of postoperative dysphagia in the MSP group was significantly higher and the difference between the two groups was statistically significant (*χ* = 6.23, *p*=0.01). Ascites was a frequently encountered complication after surgery. The incidence of ascites in the sMSP group and MSP group was 83.33% and 90.00%, respectively. Ascites usually occurred 1 week after surgery and dissipated within 2 weeks of treatment. The high-risk incident time was 1 week to 3 months after surgery. Patients generally had no abnormal discomfort. Although patients in this study were given subcutaneous low molecular weight heparin sodium and oral aspirin prophylactically after surgery, the effect was not plausible. The incidence of postoperative abdominal hemorrhage in the sMSP and MSP groups was 16.6% (5/30) and 20.0% (6/30), respectively. There was no statistically significant difference between the two groups (*χ* = 0.11, *p*=0.73). Hepatic encephalopathy and esophageal anastomotic leakage were also critical complications. Although their respective incidence was not high, the treatment was challenging and the prognosis was poor.

## 4. Discussion

Portal hypertension caused by cirrhosis is a common clinical syndrome that seriously affects the quality of life of patients. Liver transplantation is regarded as the only “curative” treatment of portal hypertension [12]; however, high medical costs and limited liver sources are two major issues associated with liver transplantation [2, 4]. Over 50% of patients in China with portal hypertension have undergone various “palliative” surgical procedures. Among them, devascularization represented by the modified Sugiura procedure has been regarded to have a satisfactory therapeutic effect [13, 14]. Unfortunately, the major concern of this procedure is high postoperative bleeding and mortality [2, 12].

**Figure 2:**
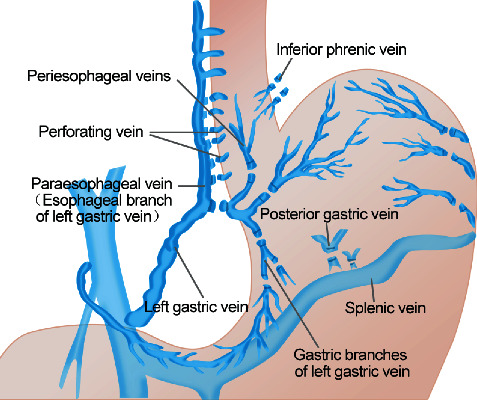
The selective modified Sugiura procedure.

**Figure 3:**
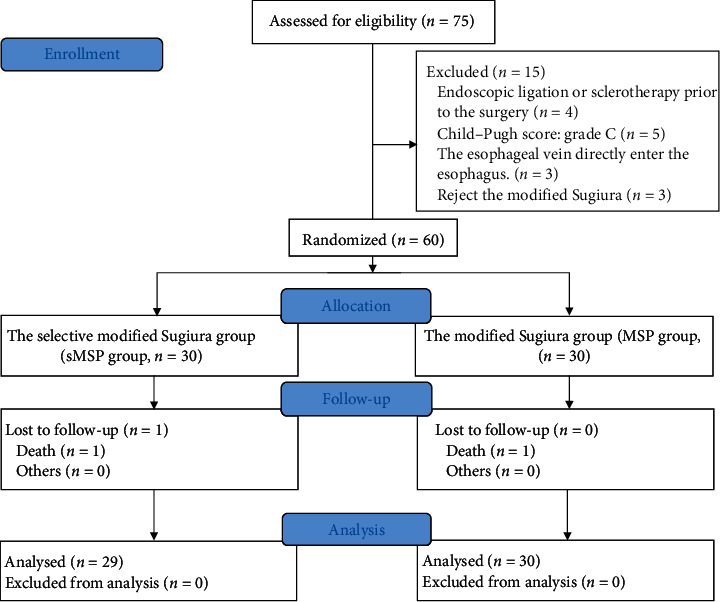
Screening and randomization of patients.

**Figure 4:**
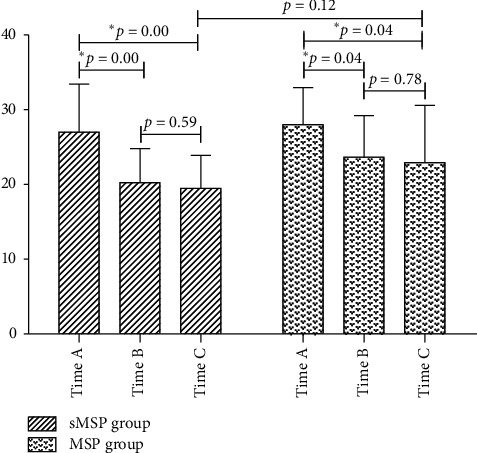
Changes in free portal venous pressure during the operation. Time A, after laparotomy, before splenectomy; time B, after splenectomy; time C, after transesophageal anastomosis. ^*∗*^The differences were statistically significant.

**Figure 5:**
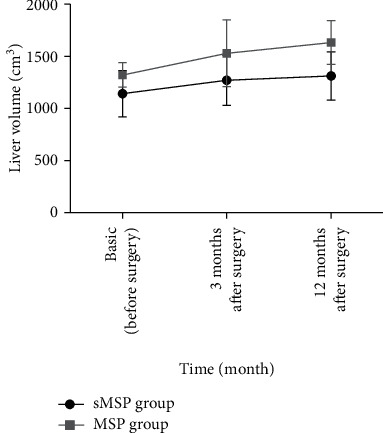
Changes in liver volume during the perioperative period.

A selective esophagogastric devascularization procedure was proposed by Yang et al. [11]. Specifically, the operation retained the esophageal branch of the left gastric vein and selectively devascularized the gastric branches of the left gastric vein, the perforating vein of the left gastric vein at of the lower end of esophagus, and the high perforating vein at the upper end of the esophagus (Figure 2). In theory, the left esophageal branch of the left gastric vein enters the thoracic cavity through the esophageal hiatus and then connects to the superior vena cava through the azygos vein to form a spontaneous portal vein shunt. Meanwhile, the esophageal branch of the left gastric vein has multiple branches connecting to the esophagus, which are named the esophageal perforating vein and the high esophageal perforating vein. These vein branches connect the esophageal epithelial venous plexus, the deep layer of lamina propria, and the paraesophageal vein. Selective retention of the esophageal branch of the left gastric vein and devascularization of the esophageal perforating vein and high esophageal perforating veins may have a certain degree of risk of maintaining the portal shunt and reducing esophageal variceal bleeding [11]. We therefore proposed selective esophagogastric devascularization in the modified Sugiura procedure which may have certain benefits to patients.

The modified Sugiura procedure is a relatively complicated operation; it mainly applies to these patients with hemorrhagic portal hypertension. Indications of the surgery mainly include the decompensated stage of cirrhosis after hepatitis, accompanied by recurrent gastrointestinal bleeding, and the bleeding should be stopped before surgery. At the same time, liver function is an important index to evaluate the feasibility of surgery; Child–Pugh C may be a contraindication for performing the surgery [2].

Our results show that selective esophagogastric devascularization in the modified Sugiura procedure can significantly improve the quality of life of patients 1 year after surgery, improve the Child–Pugh score of liver function, and reduce the incidence of postoperative dysphagia. However, different from our expectations, our findings did not show that sMSP was effective in reducing the risk of postoperative rebleeding, even though it did not increase the associated risk. In addition, we found that splenectomy had a remarkable effect on reducing FPP, while selective esophagogastric devascularization did not demonstrate a significant effect on FPP in a short term. It is unknown whether selective esophagogastric devascularization can affect FPP or how it affects FPP in a certain period after surgery. Based on the angle of portal vein pressure change, the benefit of modified Sugiura procedure or selected modified Sugiura procedure to patients was mainly obtained by splenectomy. It may have little to do with esophagogastric devascularization or esophageal transverse anastomosis. Nevertheless, it is certain that selective esophagogastric devascularization does not increase postoperative complications of the modified Sugiura procedure in the short term.

Johnson et al. [15] and Zhang et al. [16] showed that an esophageal transverse anastomosis did not reduce the incidence of complications including postoperative hemorrhage or rebleeding, but increased the risk of complications associated with the anastomosis, such as dysphagia and esophageal anastomotic fistula. In this study, 60 patients underwent successful surgical treatment. One patient developed an esophageal anastomotic fistula within 1 week after surgery and was cured after conservative medical treatment for 1 month. Another patient was not recruited into the study because of endoscopic sclerotherapy for esophageal hemorrhage. The patient developed an esophageal anastomotic fistula after the modified Sugiura procedure and died after medical treatment. Dysphagia is a common complication after surgery, and its occurrence may be closely associated with the esophageal transverse anastomosis. It is thus important to further investigate the necessity of esophageal transverse anastomosis during the modified Sugiura procedure.

For portal hypertension treatment, most treatment strategies including TIPS can be called “palliative,” except for liver transplantation. Therefore, the quality of life of patients appears to be a key indicator to assess the therapeutic effects. We showed that selective esophagogastric devascularization in the modified Sugiura procedure significantly improved the quality of life of patients 1 year after surgery. Such improvement in quality of life may be highly related to the improvement of postoperative liver function and the low incidence of dysphagia. However, it is still unclear whether the improvement in patients' quality of life was associated with the esophageal transverse anastomosis. What effects the esophageal transverse anastomosis may have on the postoperative quality of life of patients and how selective esophagogastric devascularization in the modified Sugiura procedure could affect quality of life of patients by abandoning the esophagus transverse anastomosis are questions that should be explored in the future.

In conclusion, for patients with cirrhotic hemorrhagic portal hypertension, sMSP did not reduce the incidence rate of rebleeding in the short term based our finding. However, it may lead to significantly augmented quality of life in patients, improved liver function, and reduced postoperative dysphagia in comparison with MSP.

## Figures and Tables

**Figure 1:**
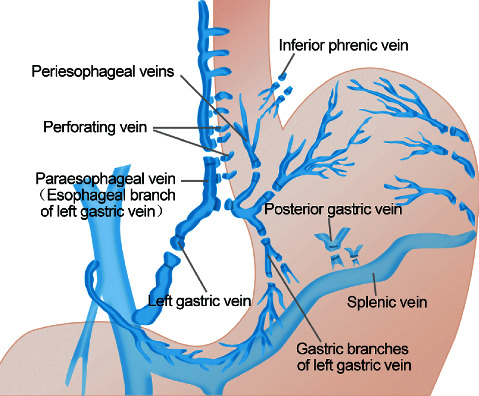
The modified Sugiura procedure.

**Table 1:** Demographic characteristics of patients.

Items	sMSP group (*n* = 30)	MSP group (*n* = 30)	*p*
Age (year)	47.15 ± 8.70	43.30 ± 12.81	0.31

Sex			
Male	21	23	0.55
Female	9	7	

History of hemorrhage before surgery			
Once	19	16	0.42
Twice	11	14	

Degree of esophageal-gastric varices			
Moderate	16	17	0.79
Severe	14	13	

Child–Pugh score			
Grade *B*	12	14	0.60
Grade *C*	18	16	

Width of portal vein (cm)	1.40 ± 0.17	1.34 ± 0.20	0.46
Blood flow velocity of portal vein	14.57 ± 3.71	12.63 ± 2.92	0.22
Free portal pressure (mmHg)	27.00 ± 6.41	28.00 ± 4.93	0.64
Liver volume (cm^3^)	1142.73 ± 221.91	1221.45 ± 117.12	0.47
Ascites	10	9	0.78
Hepatic encephalopathy	0	0	—

**Table 2:** Patients' quality of life 1 year after surgery.

Items	sMSP group		MSP group		*p*	95% CI
	Mean	SD	Mean	SD		
Physical functioning	80.14	2.555	73.38	1.609	0.00^*∗*^	5.14 to 8.37
Role-physical	64.33	5.562	62.15	6.189	0.29	−1.99 to 6.35
Bodily pain	68.86	4.316	69.62	5.546	0.65	−4.21 to 0.70
General health	72.62	3.801	65.69	5.483	0.00^*∗*^	3.68 to 10.16
Vitality	60.19	5.419	59.00	6.403	0.56	−2.98 to 5.36
Social functioning	76.00	3.578	70.62	2.815	0.00^*∗*^	3.00 to 7.76
Role-emotional	58.76	4.560	59.69	4.854	0.57	−4.28 to 2.42
Mental health	70.29	3.349	61.77	4.024	0.00^*∗*^	5.91 to 11.11

**Table 3:** Complications after surgery.

Complications	sMSP group （*n* = 30）	MSP group （*n* = 30）	*χ*	*p*
Ascites	25	27	0.57	0.44
Portal thrombosis	9	11	0.58	0.78
Splenic vein thrombosis	4	4	0.00	1.00
Dysphagia	5	14	6.23	0.01^*∗*^
Abdominal bleeding	5	6	0.11	0.73
Esophageal anastomotic leakage	0	1	1.01	0.31
Hepatic encephalopathy	0	1	1.01	0.31
Death	1	0	1.01	0.31

## Data Availability

The data used to support the findings of this study are available from the corresponding author upon request.

## Abbreviations

MSP group: Modified Sugiura group
sMSP group: Selective modified Sugiura group
WMD: Weighted mean difference
CI: Confidence interval
TIPS: Transjugular intrahepatic portal systemic shunt
FPP: Free portal venous pressure
CT: Computational tomography.
